# The burden of cutaneous fungal infections in a tertiary care hospital in Pakistan

**DOI:** 10.12669/pjms.41.1.9061

**Published:** 2025-01

**Authors:** Najam-us-Saher, Saadia Tabassum, Madiha Sajid, Yumna Khabir, Aisha Faheem

**Affiliations:** 1 Najam-us-Saher, MBBS, FCPS Assistant Professor Dermatology, The Aga Khan University Hospital, Karachi Pakistan; 2 Saadia Tabassum, MBBS, FCPS Assistant Professor Dermatology, The Aga Khan University Hospital, Karachi Pakistan; 3 Madiha Sajid, MBBS, FCPS Assistant Professor Dermatology, Dow International Medical, College and Dow University of Health Sciences, Karachi Pakistan; 4 Yumna Khabir 3rd year MBBS Student, Dow Medical College; 5 Aisha Faheem, M.Sc. (Biotechnology), MBA (Pharmaceutical Management) Research Assistant, Dow Research Institute of, Biotechnology and Biomedical Sciences, Karachi Pakistan

**Keywords:** Dermatology, Fungal infection, Tinea cruris, Health, Disease

## Abstract

**Objective:**

In Pakistan, the real extent of fungal infection is unknown. Our objective was to estimate the burden of major fungal diseases here to emphasize their public health importance.

**Methods:**

In this retrospective study, the medical records (January 2019 - December 2019) of 863 patients diagnosed with superficial fungal infection (SFI) were reviewed at the dermatology department of Aga Khan University Hospital (AKUH) Karachi. Stata 17.0 was used to analyze the data. Chi-squared test or Fisher’s exact test was used to evaluate association between variables. Descriptive analyses include means, frequencies, standard deviations, and percentage tests whereas Poisson regression with robust standard error was used to examine the association of age, gender, diabetes, and type of SFI with recurrence of SFI.

**Results:**

In this study, the most common SFI was tinea cruris (234/863, 27.1%) in men (n=62, 21.7%) and females (n=172, 29.8%) followed by pityriasis versicolor (n=77, 26.9%) in males and tinea corporis (n=115, 19.9%) in females. Tinea cruris recurrence rates were similar between males and females (34/234, 14.5%).

**Conclusion:**

These findings show that fungal infections are a public health problem in Pakistan and that additional research is needed to assess their frequency in the general population. Clinicians must be taught and made aware of these infections to enhance diagnosis and treatment.

## INTRODUCTION

Cutaneous fungal infections are superficial infections typically involving the skin, hair, and nails.^1^ They constitute a significant global problem with a prevalence of about 20–25% of the world’s population, making these one of the most frequent forms of cutaneous infection 2 and the 4th most frequent problem worldwide after headache and dental caries 3.Cutaneous fungal infections are commonly caused by dermatophytes and additionally by non-dermatophyte fungi and yeast, commonly the Candida and Malassezia species.^1^,^4^ Dermatophytic infections comprise most of the cutaneous fungal infections with Trichophyton, Epidermophyton, and Microsporum species being the common pathogens with some variations according to geographic location.^5^ The term dermatophytes refer to fungal organisms that causes the tinea subgroup of infection^6^, which are further classified by the region of the body infected (e.g., tinea pedis, tinea corporis, tinea cruris, tinea manuum, tinea capitis).^1^ Tinea unguium, also known as onychomycosis, is caused most frequently by dermatophytes, but non-dermatophytes and Candida species also can cause it.

Dermatophytes thrive at 25-28°C and therefore, dermatophytic infections are more common in tropical countries. The factors which can lead to exacerbation or recurrence of preexisting fungal infection include warm and humid environments, wearing occlusive clothing and shoes, living in congested environments causing skin-to-skin contact, and petting.^2^ Additional factors more prevalent in developed countries are increased facilities of contact sports like swimming, increasing prevalence of diabetes mellitus and arteriovenous diseases, and increased geriatric population.^7^ Furthermore, owing to increased tourism, migration, and international sports activities the geographic predisposition of cutaneous fungal infections is changing, and uncommon species are identified in several cases.^2^

Potassium hydroxide (KOH) preparation and fungal culture of cutaneous scrapings taken from the infected areas are the most used diagnostic tests for the diagnosis of superficial fungal infections. These often produce false-negative results, resulting in a diagnostic dilemma.^8^ However, the literature revealed that calcofluor white stain provides a quicker and more reliable method for the identification of superficial fungal infections.^9^

Knowledge of current epidemiological trends in the incidence of cutaneous fungal infections is important in diagnosis and treatment of the disease.^10^ The distribution of dermatophytes also greatly varies around the world.^11^ It is important to note that the clinical type and the causative species of superficial fungal infections also vary with geographic region, socioeconomic conditions, and habits. Some species of fungi exhibit worldwide distribution, whereas others are restricted to certain continents or geographic regions.^2^

It has been observed that there is an increase in the number of patients presenting with superficial fungal infections in dermatology clinics, with a sizeable number of those coming with repeated infections. Moreover, there is scarcity of data on epidemiology of fungal infections from Pakistan. This study helped us to determine the burden and the present trend of various cutaneous fungal infections in a tertiary care hospital of Pakistan. Furthermore, this study will provide the researchers with baseline data for future studies on superficial cutaneous fungal infection.

## METHODS

This was a retrospective study that was carried out at the Department of Dermatology, Aga Khan University Hospital (AKUH) Karachi between January, 2019 - December 2019. Files of 863 patients presenting in the Dermatology outpatient department of AKUH from January 2019 to December 2019 were reviewed by the investigator to obtain the relevant clinical information through a history of medical records, doctor’s notes, nurse’s notes, assessment forms and relevant investigations. A code was assigned to each medical record number, used for identification of the patients. These were clinically diagnosed patients having superficial dermatophytic/yeast cutaneous fungal infections with or without microbiological evaluation of fungal scrapings or consistent histologic features of skin biopsy specimens. Patients presenting with other skin diseases were excluded from the study. Data was entered on an online form for further analysis.

### Ethical Approval:

Permission was sought from the Ethics and Review Committee on 16^th^ February 2020, under number 2020-3310-8488.

The data was analyzed using Stata 17.0. The association between various categorical variables will be investigated using the Chi-squared test or Fisher’s exact test, as appropriate. Bonferroni correction for multiple comparisons will be performed where necessary. Descriptive analyses include means, frequencies, standard deviations, and percentage tests; whereby mean and standard deviation will be used for continuous variables (i.e., age, education); frequency and percentage will be used for categorical variables (gender, affected part of the body, and comorbids). Poisson regression with robust standard error was used to examine the association of age, gender, diabetes, and type of SFI with recurrence of SFI.

## RESULTS

Superficial fungal infections of the skin, hair and nail spare no age, gender and race. Owing to frequent presentation of patients with SFI in the dermatology clinics, we aimed to determine the rates of occurrence and recurrence of various clinical presentations of SFIs. In this study, the mean age of the patients at the time of presentation was 34.85 ± 17.81 years. Two thirds of the SFI patients were females (577, 66.9%). The most common comorbidities among patients were hypertension (13.7%), diabetes (11%), malignancy (1.5%) and immunosuppression (0.9%). The most affected part of the body was trunk (51%) followed by genitals (25.5%), feet (15.6%), face (13.6%), nails (13.1%), hands (9.6%) and scalp (8.8%) (Table-I).

**Table-I:** Baseline demographic and clinical characteristics.

Characteristic	Frequency	%
Age at presentation, years*		
Mean ± SD	34.85 ± 17.81	
Median (min - max)	32 (0.1-91)	
***Gender***		
Male	286	33.1
Female	577	66.9
***Age categories***		
<18	113	13.1
18-29	264	30.6
30-49	303	35.1
≥ 50	183	21.2
***Comorbid***		
Diabetes	95	11.0
Hypertension	118	13.7
Hyperthyroidism	3	0.3
Hypothyroidism	8	0.9
Malignancy	13	1.5
Immunosuppression	8	0.9
Asthma	8	0.9
Mental health + brain disorders	8	0.9
Other skin diseases	6	0.7
Other	18	2.1
***Affected part of the body***		
Trunk	441	51.0
Feet	135	15.6
Nails	113	13.1
Genitals	220	25.5
Hands	83	9.6
Scalp	76	8.8
Face	117	13.6

Tinea cruris was the most common SFI (234/863, 27.1 %) followed by tinea corporis (18.8 %), pityriasis versicolor (18.5 %), tinea unguium (13.6 %), tinea pedis (8.3%), candida intertrigo (4.5%), tinea capitis (3.5%) and genital candidiasis (2.7%). The most common fungal infection in men and women were pityriasis versicolor (77/286, 26.9%) and tinea cruris (172/577, 29.8%) respectively. Tinea pedis (p<0.001) and pityriasis versicolor (p< 0.001) were significantly more common in males than females whereas genital candidiasis was significantly prevalent in females (p=0.003) (Table-II). Tinea cruris was the most common SFI in patients aged 18-29 (79/264, 29.9%) and 30-49 (86/303, 28.4%) years. By contrast, pityriasis versicolor and tinea unguium were the most common SFI among those aged < 18 years (27/113, 23.9%) and ≥ 50 years (53/183, 29%), respectively (Table-III).

**Table-II:** Distribution of various superficial fungal infections according to gender.

Gender							

	Count	Male	Female	Total	Count	%	P*

		(n=286)	(n=577)	(n=863)			

		%	Count	%			
Tinea cruris	62	21.7	172	29.8	234	27.1	0.011
Tinea corporis	47	16.4	115	19.9	162	18.8	0.216
Pityriasis ersicolor	77	26.9	83	14.4	160	18.5	<0.001
Tinea unguium	27	9.4	90	15.6	117	13.6	0.013
Tinea pedis	46	16.1	26	4.5	72	8.3	<0.001
Candida intertrigo	7	2.4	32	5.5	39	4.5	0.039
Tinea capitis	13	4.5	17	2.9	30	3.5	0.227
Genital andidiasis	1	0.3	22	3.8	23	2.7	0.003
Tinea faciei	6	2.1	12	2.1	18	2.1	0.986
Tinea incognito	5	1.7	8	1.4	13	1.5	0.768
Tinea manuum	3	1.0	6	1.0	9	1.0	>0.999
Oral candidiasis	1	0.3	2	0.3	3	0.3	>0.999
Tinea imbricata	1	0.3	1	0.2	2	0.2	0.553
Anal candidiasis	1	0.3	0	0.0	1	0.1	0.331

All p* values < 0.0035 are significant.

**Table-III:** Distribution of various superficial fungal infections according to age.

Age Categories								

	<18		18-29		30-49		≥ 50	

	Count	%	Count	%	Count	%	Count	%
Tinea cruris	17	15.0	79	29.9	86	28.4	52	28.4
Tinea corporis	17	15.0	57	21.6	61	20.1	27	14.8
Pityriasis versicolor	27	23.9	66	25.0	55	18.2	12	6.6
Tinea unguium	5	4.4	19	7.2	40	13.2	53	29.0
Tinea pedis	7	6.2	17	6.4	31	10.2	17	9.3
Candida intertrigo	6	5.3	8	3.0	15	5.0	10	5.5
Tinea capitis	23	20.4	4	1.5	3	1.0	0	0.0
Genital candidiasis	3	2.7	10	3.8	6	2.0	4	2.2
Tinea facei	7	6.2	4	1.5	5	1.7	2	1.1
Tinea incognito	1	0.9	4	1.5	3	1.0	5	2.7
Tinea mannum	1	0.9	1	0.4	6	2.0	1	0.5
Oral candidiasis	0	0.0	0	0.0	1	0.3	2	1.1
Tinea imbricata	0	0.0	1	0.4	1	0.3	0	0.0
Anal candidiasis	0	0.0	0	0.0	0	0.0	1	0.5

**Fig.1:**
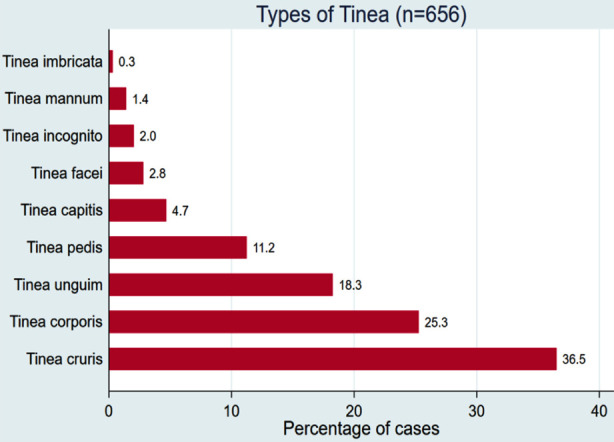
Types of dermatophytoses / tinea infections.

**Fig.2:**
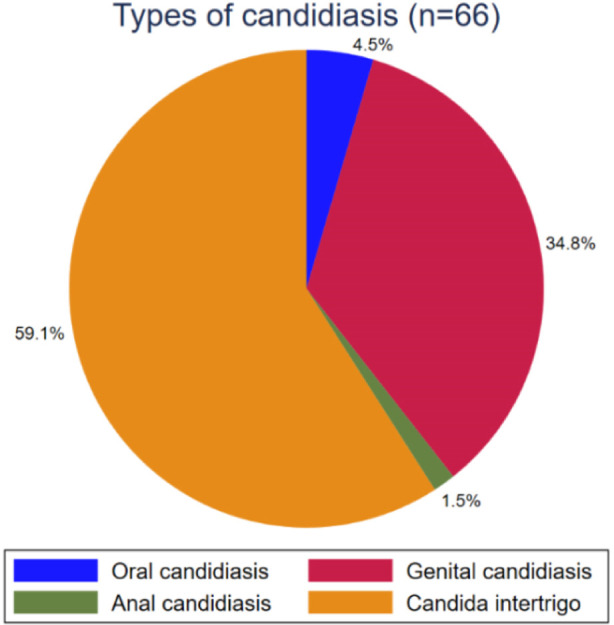
Prevalence of various types of candidiasis.

Tinea cruris accounted for 36.5% (234/656) of all dermatophytosis cases, followed by tinea corporis (25.3%), tinea unguium (18.1%), tinea pedis (11.2%) and tinea capitis (4.7%) (Fig.1). Among candidiasis (n=66), the most common clinical forms were candida intertrigo (59.1%), followed by genital candidiasis (20.5%), oral candidiasis (4.5%) and anal candidiasis (1.5%) (Fig. 2).

Overall, the rate of recurrence of SFI was 10.2% (88/863). The mean time of recurrence was 10.41 ± 19.53 months. Recurrence rates were similar between males and females. Individuals aged ≥ 50 were more likely to have had a recurrence compared with other age groups. The most recurring SFI was Tinea cruris (34/234, 14.5 %) (Table-IV).

**Table-IV:** Recurrence rates among the SFI patients (n= 863).

	Number of SFI patients	Number (%) of individuals with recurrence
All	863	88 (10.2)
***Age, years***		
<18	113	10 (8.8)
18-29	264	24 (9.1)
30-49	303	27 (8.9)
≥ 50	183	27 (14.8)
***Gender***		
Male	286	30 (10.5)
Female	577	58 (10.1)
***Type of SFI***		
Tinea cruris	234	34 (14.5)
Tinea corporis	162	21 (13.0)
Pityriasis versicolor	160	7 (4.4)
Tinea unguium	117	10 (8.5)
Tinea pedis	72	8 (11.1)
Candida intertrigo	39	3 (7.7)
Tinea capitis	30	4 (13.3)
Genital candidiasis	23	3 (13.0)

A review of the determinants of recurrence of SFI is shown in Table-V through a univariate and multivariable analysis. Age, which was significantly associated with recurrence in the univariate analysis (p=0.033), lost statistical significance in the multivariable analysis (p=0.147). In the multivariable analysis, patients with pityriasis versicolor were significantly less likely to have a recurrence compared with those with dermatophytosis (p=0.012).

**Table-V:** Determinants of SFI recurrence (n=863).

	Univariate analysis		Multivariable analysis	

	Incidence rate ratio (95% CI)	P	Incidence rate ratio (95% CI)	P
Age, years	1.01 (1.001, 1.02)	0.033	1.01 (0.997, 1.02)	0.147
***Gender***				
Male	1.04 (0.69, 1.58)	0.842	1.13 (0.74, 1.72)	0.582
Female	1.00			
***Diabetes***				
Yes	1.28 (0.72, 2.26)	0.402	0.97(0.50, 1.87)	0.926
No	1.00		1.00	
***Type of SFI***				
Dermatophytosis	1.00		1.00	
Candidiasis	0.94 (0.45, 1.96)	0.872	0.95 (0.46, 1.98)	0.896
Pityriasis versicolor	0.33 (0.15, 0.74)	0.007	0.35 (0.15, 0.79)	0.012
Co-infection	1.72 (0.29, 10.08)	0.547	1.46 (0.22, 9.51)	0.691

## DISCUSSION

Fungi are rampant in our country due to warm and humid climate and so are fungal infections. In our study middle aged females of ages 30-49 (35.1%) were most commonly affected, and on the whole females made up two-thirds of the subjects (66.9%), despite having less comorbid states than male counter parts included in the study. While, according to a study reporting a global burden of superficial fungal infections young males were more affected.^12^ This difference in gender prevalence, could be due to hot, humid weather along with heavy clothing, indoor lifestyles, and lack of proper hygienic measures. This also showed that SFIs are prevalent in even immunocompetent patients.

In this study, the most prevalent SFI was tinea cruris (27.1%), followed by tinea corporis (18.8%), pityriasis versicolor (18.5%), tinea unguium (13.6%), tinea pedis (8.3%), candida intertrigo (4.5%), tinea capitis (3.5%), genital candidiasis (2.7%) and others. On the other hand, a study conducted in India stated, tinea corporis (78.1%) as the commonest clinical presentation, followed by tinea cruris (10.1%) tinea manuum (2.5%), tinea faciei (1.8%), and tinea pedis (0.7%).^13^ Low number of tinea capitis in our study could be due to fewer number of children in the study, as it is the commonest pattern in this age group.

In this study tinea cruris was most prevalent in both males (21.7%) and females (29.8%) followed by P. versicolor (26.9%) in males and T. corporis (19.9%) in females. On the other hand, a study from Bangladesh showed highest 67.65% tinea pedis in males and faciei being the least common presentation in 20% males while in female group highest 80% were infected by Tinea faciei and lowest 32.35% were infected by Tinea pedis.^14^ Overall, the rate of recurrence of SFI was 10.2%. This could be due to emerging resistance which is a critical issue encountered during treatment. Recently chronic, extensive, and recalcitrant cutaneous fungal infections are being reported.^15^

This study also showed that 26.1% of the superficial fungal infections were caused by non-dermatophytes with 18.5% by malasezzia species. Although superficial infections are not associated with the mortality, they are greatly affecting a patient’s psychosocial life, compromising the quality of life.^16^ Onychomycosis, accounting for 13.6% of the patient can affect routine physical activities and pose cosmetic problems.

It is noticeable from our study that there is an upsurge in both dermatophytic and non-dermatophytic infections of the skin and they alone pose a considerable burden on the health care system. From this study it can also be deduced that there is an increasing trend of not only naive but also recurrent superficial fungal infections of the skin in the urban immunocompetent population despite them having better health care services, hygiene and sanitary conditions.

A study from Lahore, Pakistan and India reported 34.80% and 20% prevalence of fungal skin infections respectively.^17^,^18^ Concomitantly, following the results and trends of this study, demands further prospective local research to highlight the escalating burden and identify prevalence of various dermatophytic and non dermatophytic species and hence bring innovation in the treatment to combat the growing resistance.

### Limitations:

We were unable to follow our patients, as the data was collected retrospectively. In addition, no data was collected from Pakistan’s rural areas.

## CONCLUSION

Superficial fungal infections are common with wide and sometimes challenging differential diagnosis. Physicians must be knowledgeable with this condition to provide an accurate diagnosis and commence appropriate therapy. In Pakistan, fungal infections are widespread. The current study is crucial not only for Pakistan’s policy-making strategic groups but will also help strategic organizations worldwide to design strategies to avoid the future development of cutaneous fungal diseases not only in urban regions, but also in rural areas.

For decades, the increased burden of cutaneous fungal infections has been a stubbornly ignored public health issue. One of our study’s drawbacks is that no data was collected from Pakistan’s rural areas. One key possibility is a lack of resources for the general people in remote locations for prospective diagnostic testing or appropriate treatments. Continued efforts are needed at many levels across the world to contribute to better fungal diagnostic methods to guide early intervention and management, as well as to prevent resistance by restricting the use of anti-infective medicines, especially antifungals.

### Authors Contribution:

**NS:** Conceived, designed, and did editing of manuscript, is responsible for integrity of research.

**MS** & **AF:** Literature search, manuscript writing and editing.

**YK:** Did critical review, statistical analysis and interpretation of data.

**ST:** Literature search, critical review.

All authors have approved the final manuscript and are accountable for integrity of the study.
